# Salvinorin A Administration after Global Cerebral Hypoxia/Ischemia Preserves Cerebrovascular Autoregulation via Kappa Opioid Receptor in Piglets

**DOI:** 10.1371/journal.pone.0041724

**Published:** 2012-07-24

**Authors:** Zhenhong Wang, Nan Ma, John Riley, William M. Armstead, Renyu Liu

**Affiliations:** 1 Department of Anesthesiology and Critical Care, Hospital of University of Pennsylvania, Philadelphia, Pennsylvania, United States of America; 2 Department of Anesthesiology, School of Medicine, Renji Hospital, Shanghai Jiaotong University, Shanghai, China; Massachusetts General Hospital, United States of America

## Abstract

**Background:**

Cerebral hypoxia/ischemia (HI) is not uncommon during the perinatal period. If occurring, it can result in severe neurologic disabilities that persist throughout life. Salvinorin A, a non-opioid Kappa opioid receptors (KOR) selective agonist, has the potential to address this devastating situation. We have demonstrated that salvinorin A administration before HI, preserves pial artery autoregulative function through both the KOR and extracellular signal-regulated kinases (ERK) pathways. In the present study, we tested the hypothesis that administration of salvinorin A after HI could preserve cerebral autoregulation via KOR and ERK pathway.

**Methodology/Principal Findings:**

The response of the pial artery to hypercapnia, hypotension and isoproterenol were monitored before and 1 hour after HI in piglets equipped with a cranial window. Four groups of drug administration were performed after HI. The control group had DMSO (1 µl/kg, i.v.) administrated immediately after HI. Two salvinorin A treated groups had salvinorin A (10 µg/kg, i.v.) administrated 0 and 30 min after HI, respectively. The 4^th^ group had salvinorin A and the KOR antagonist norbinaltorphimine (Nor-BIN, 1 µM topical) co-administrated 0 min after HI (n = 5). The dilation responses of the pial artery to hypercapnia and hypotension were impaired after global HI and were preserved with salvinorin A administration immediately or 30 min after HI. The preservation of autoregulation was abolished when nor-BIN was administered. Levels of phosphor-ERK(pERK)/ERK in the cerebrospinal fluid (CSF) were measured before and 1 hour after HI. After HI, the pERK/ERK levels significantly increased in both DMSO control group and salvinorin A and nor-BIN co-administration group. The elevated levels of pERK/ERK were not observed with salvinorin A only groups.

**Conclusions:**

Salvinorin A administration 0 and 30 min after HI preserves autoregulation of pial artery to hypercapnia and hypotension via kappa opioid receptor and ERK pathway.

## Introduction

The neuronal death and behavioral dysfunction caused by hypoxia/ischemia (HI) induced brain injury is not uncommon during the perinatal period. Worldwide, 23% of all new birth deaths are associated with asphyxia and 30% of children who’s births involved moderate hypoxic–ischemic encephalopathy (HIE) may develop mental retardation, learning difficulties, and other disabilities [1]. Unfortunately, there is no medication available to manage this devastating situation.

Kappa opioid receptors (KOR), a subfamily of inhibitory regulative G-protein coupled receptors, are known to be integral to cerebral neuroprotection in cell and animal models [2], [3], [4]. Treatment with KOR agonists prolongs animal survival rates after cerebral ischemia [5]. It reduces the neuronal necrosis region [6] and infarction induced by cerebral ischemia [7], and it improves the motor and memory functions after cerebral ischemia [8]. Downstream of the KOR response, extracellular signal-related kinase (ERK) signaling is believed to contribute to the subsequent neuron damage and cell death [9], [10] as the level of ERK activity is upregulated in the cerebrospinal fluid (CSF) after HI [11].

KOR agonists cause dilation of cerebral vessels [12], a key feature required to maintain cerebral autoregulation and reduce brain injury from ischemia. After ischemia, cerebral autoregulation is impaired, resulting in decreased cerebral blood flow and neuron death [13], [14], [15]. We have demonstrated in a piglet model that administration of salvinorin A before global HI preserved the autoregulation of the cerebral artery, an important mechanism for the preservation of neuronal integrity [16]. In the current study, we hypothesized and tested whether administration of salvinorin A after HI could preserve cerebral autoregulation via the KOR and ERK pathway.

## Results

### Salvinorin A Preserves Pial Artery Autoregulation to Hypercapnia after Global Cerebral HI

**Figure 1:**
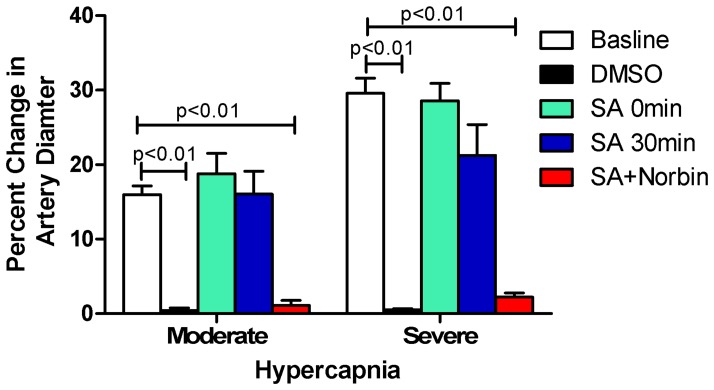
Effects of post HI salvinorin A administration on pial artery dilation to hypercapnia. HI with DMSO impaired dilation of pial artery to hypercapnia. SA administrated at onset and 30 min after HI preserved the dilation of pial artery to moderate and severe hypercapnia, which were blunted by norbinaltorphimine (Norbin). N = 5 in each group. Percentage change =  (diameter after hypercapnia−diameter before hypercapnia)/diameter before hypercapnia)*100. SA: Salvinorin A; Moderate: hypercapnia with PaCO_2_ of 50 to 60 mmHg; Severe: hypercapnia with PaCO_2_ of 70 to 80 mmHg.

As shown in figure 1, the small pial artery dilated in response to two levels of hypercapnia before HI (presented as baselines). The dilatation responses to hypercapnia were blunted after HI when DMSO was administrated immediately at the end of HI (ps<0.01 as compared with the baselines before HI). Administration of salvinorin A (10 µg/kg), immediately or 30 min after HI, preserved the dilation responses of pial artery to hypercapnia. Such responses were abolished completely when salvinorin A and norbinaltorphimine (nor-BIN) were co-administrated 30 min after HI (ps<0.01 as compared with baselines before HI). Similar observations were obtained in pial arterioles (data not shown).

### Salvinorin A Preserves Pial Artery Autoregulation to Hypotension after Global Cerebral HI

**Figure 2:**
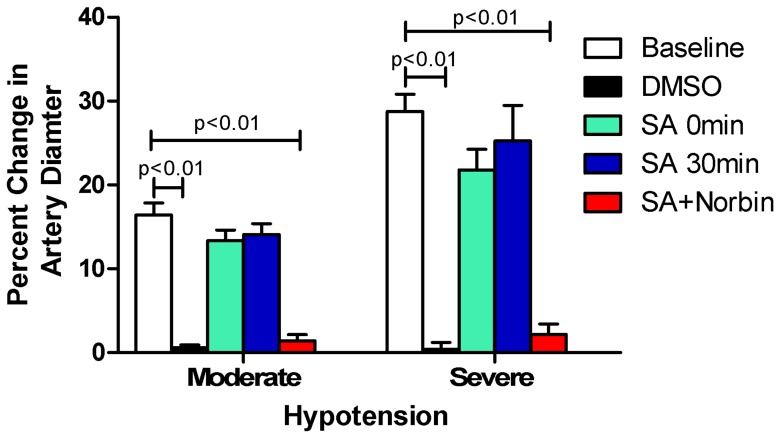
Effects of post HI salvinorin A administration on pial artery dilation to hypotension. HI with DMSO damaged dilation of pial artery to hypotension. SA administrated at onset and 30 min after HI preserved the dilations of pial artery to moderate and severe hypotension, which were blunted by co-administration of norbinaltorphimine (Norbin). Percentage change =  (diameter after hypotension–diameter before hypotension)/diameter before hypotension)*100. N = 5 in each group. SA: Salvinorin A. Moderate: 25% decrease of mean blood pressure. Severe: 45% decrease of mean blood pressure.

Similar to the result of hypotension, the small pial artery dilated in response to two levels of hypotension before HI hypercapnia (presented as baselines, figure 2) before HI. The dilatation responses were blunted after HI when DMSO was administrated immediately at the end of HI (ps<0.01 as compared with the baselines before HI). Salvinorin A (10 µg/kg), administrated immediately or 30 min after HI, preserved the dilation responses of pial artery to hypotension. Such responses to hypotension were abolished when salvinorin A and nor-BIN were co-administrated 30 min after HI (ps<0.01 as compared with the baselines). Similar observations were obtained in pial arterioles.

### Pial Artery Responses to Isoproterenol Remain Unchanged in all Sets of Experiments

**Figure 3:**
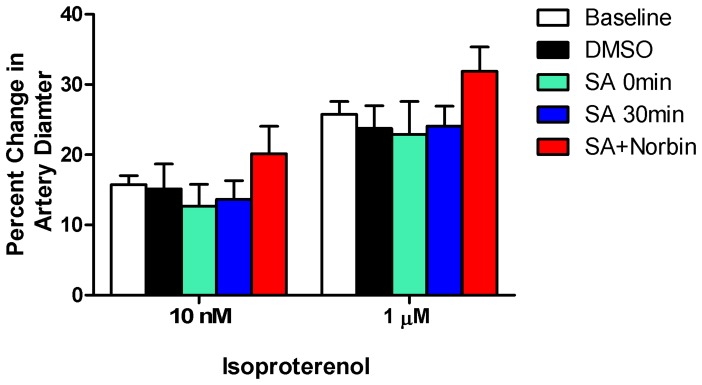
Isoproterenol induced artery dilation was independent of KOR or ERK signaling. Effects of isoproterenol (10 nM, 1 µM) on pial artery diameter before (baseline) and after HI did not change significantly in the presence and absence of various interventions, compared to baseline p>0.05. N = 5 in each group. Percentage change =  (diameter after isoproterenol−diameter before isoproterenol)/diameter before isoproterenol)*100; SA: Salvinorin A; Norbin: norbinaltorphimine.

As a positive control, pial artery responses to isoproterenol (indicated in figure 3) were measured and no change was observed among all groups before and after HI.

### ERK Signaling is Involved in the Preservation Effects of Salvinorin A

**Figure 4:**
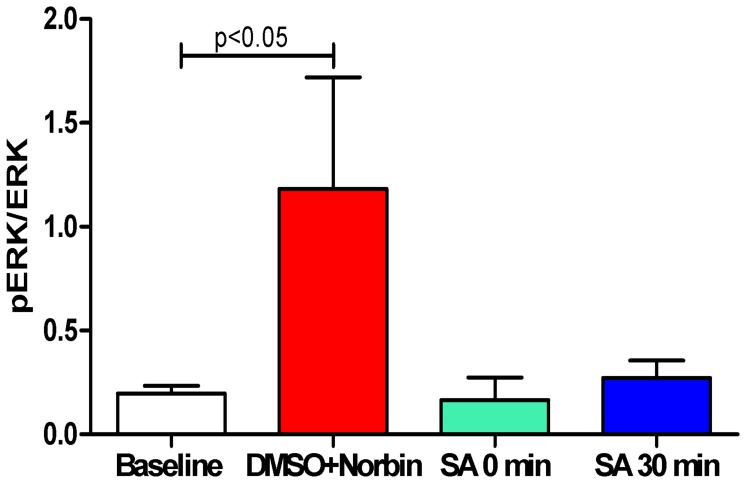
Salvinorin A administration blocked the elevated CSF ERK activity observed 1 h after HI. The ration of pERK/ERK at 1 hour after HI in the control groups (n = 10, DMSO and nor-BIN groups) increased significantly compared with the baseline. The baseline for all the groups are pulled together (n = 20) and the data from DMSO and nor-BIN groups were pulled together and presented as DMSO+Norbin (n = 10) to increase the power of the statistical analysis because of some large variances were observed. The elevated ERK activities were abolished in the groups with salvinorin A administrated immediately (n = 5) or 30 min (n = 5) after HI. Norbin: norbinaltorphimine; SA: Salvinorin A.

The ERK activities are quantified as the ratio of pERK/ERK levels in CSF. The ERK activity data in groups without salvinorin effects (DMSO group and SA+Norbin group; renamed as DMSO+Nornin group) are combined. As indicated in figure 4, the ERK activity in groups without salvinorin increased significantly 60 min after HI (p<0.05 as compared with pre-HI baseline). The ERK activity of salvinorin A administration groups reduced to the baseline level.

## Discussion

There are three new findings from this study. First, administration of salvinorin A instantly or 30 minutes after HI preserves the pial artery dilation response to hypercapnia and hypotension. Second, the preservation effects of salvinorin A were blunted KOR antagonist, nor-BIN. And third, salvinorin A blocked the increase of CSF ERK activity after HI.

### The Problem of Cerebral HI and the Potential Role of KOR Agonist

Both birth asphyxia and pediatric ischemic stroke are the common complications of childbirth. Perinatal HI, occurring in both these complications, can induce severe and permanent neuropsychological deficits, including delayed cognitive and behavioral development, mental retardation, cerebral palsy, and epilepsy, which is devastating for the patients, the families, and society [17], [18]. Unfortunately, there is no medication available for effective perinatal HI management. Hypothermia is the only treatment for HIE to reduce negative complications. But it is not widely accepted in clinical practice or recommended to combine with pharmacologic agents [19]. Recombinant tissue type plasminogen activator (t-PA), a FDA approved treatment for acute ischemic stroke, showed adverse effects including increased stroke infarct volume in mice subjected to induced stroke [20] and impaired cerebral hemodynamics [21].

The neuroprotective effects of KOR agonists have been demonstrated in other ischemia animal models. For example, KOR agonist BRL 52537 and CI-977 reduces cortical damages, including brain swelling and infarction volumes, in response to different levels of ischemia when administrated 30 min before [22] or up to 6 hours after the insult [23]. These findings suggest that KOR agonists could be a valuable alternative medication for HI treatment in clinical settings.

### Salvinorin A as a Novel Medication

Although KOR agonists exhibit tremendous therapeutic value, most KOR agonists have not been used in clinical settings because of their intrinsic characteristics as opioids, (low selectivity and/or lack of an acceptable safety profile). Unlike other opioid KOR agonists, salvinorin A is the most potent, highly selective, and the only non-opioid KOR agonist known to date derived from natural sources [24], [25]. Salvinorin A is the active component of *Salvia Divinorum*, a naturally abundant perennial herb that has been consumed by humans for recreational and sacred purposes for several centuries. Many intrinsic characteristics of this compound make it a potential therapeutic medication for various neurological conditions [26], [27], [28], [29]. Those characteristics include its ability to be extracted and purified from an abundant plant or produced via easy synthesis, a rapid onset of action, lipid solubility, easy passage through the blood brain barrier, sedative and antinociceptive effects (needed features for the critically ill patient), negative pathological findings in vital organs with high dose or prolonged exposure (non-toxic), no respiratory depression and no frank hallucinatory or dysphoric effects [30]. Salvinorin A has been evaluated as a potential medication for depression [25], [31]. In addition to our previous findings demonstrating the protective effect of salvinorin A administration before HI insult, we have now demonstrated that salvinorin A administration (up to 30 min) after HI insult preserved the autoregulation of pial artery. This protective effect was abolished by the addition of the KOR antagonist, nor-BIN, which indicates that the protective effect of salvinorin A is mediated via KOR. Various studies have proven that autoregulation, a key protective mechanism of the brain [13], tends to worsen after cerebral HI [13], [14], [15]. Autoregulation of cerebral vascular tone is a key protective mechanism of the brain [13]. And impaired compensatory cerebrovasodilation during hypotension contributes to worsened outcome in the setting of ischemic stroke [32].

### The Role of ERK Signaling

ERK signaling stimulated by cerebral ischemia/reperfusion is a crucial pathway for HI injury [33]. We have demonstrated that ERK activity increases after HI [34] and the increase relates to neuronal impairment of HI [35], [36], and inhibition of such an increase is associated with neuroprotective responses against ischemia [36], [37], [38], [39], which may be associated with the reduction in apoptosis [36]. In the present study, HI induced increases in the CSF ERK activities were blocked by salvinorin A, which promoted protection of cerebral autoregulation post insult. It is worth noting that the role of ERK signaling in HI may be different before and after HI insult. Activation of ERK signaling may be related to the protective effects of preconditioning. Upregulating ERK signaling induced by preconditioning reduces neuronal apoptosis in stroke [40] and activation of ERK signaling in the hippocampus after sublethal ischemia correlates with neuroprotection induced by preconditioning [41]. Our previously published work demonstrating that pre-injury administration of salvinorin A is protective of the impairment of cerebral autoregulation post insult is consistent with these observations [16].

### Limitations

One major limitation of the present study is that more time points should be adopted to reflect the effective time window of salvinorin A administration. Future studies will be needed to demonstrate the protective effects of salvinorin A on neurological function and to elucidate the molecular mechanism for protection induced by salvinorin.

### Conclusions

Salvinorin A administration 0 and 30 min after HI preserves autoregulation of the pial artery to hypercapnia and hypotension via KOR and the ERK pathway in a piglet model.

## Materials and Methods

Salvinorin A (purity ≥98%) was obtained from ChromaDex, Inc. (Irvine, CA, USA). Isoproterenol (ISO), nor-binaltorphimine (Nor-BIN) were obtained from Sigma-Aldrich (MO, St. Louis, MO, USA). All other chemicals (reagent grade) were obtained from Sigma as well.

### Animal and Surgery for Closed Cranial Window

Newborn pigs (1–5 days old, 1.2–1.5 kg) of both sexes were used in this study. The animal experimental protocol was approved by the Institutional Animal Care and Use Committee of the University of Pennsylvania. As described previously [16], Isoflurane (1 to 2 minimum alveolar concentration) was initially used for anesthesia induction, followed by α-chloralose (30–50 mg/kg supplemented with 5 mg/kg/hr intravenously) for maintenance of anesthesia. After tracheotomy, the animals were initially ventilated with room air and kept warm with heating pad to maintain the rectal temperature at 37–39°C. Bilateral femoral arteries were catheterized to monitor the blood pressure, blood gas tensions and pH. The femoral vein was catheterized for medication administration. A closed cranial window, consisting of a steel ring with a glass cover slip, connecting to 3 ports, was placed for direct pial artery visualization and diameter measurement. Small pial arteries (120 to 160 µm) and arterioles (50 to 70 µm) were identified under a microscope, visualized on a monitor connected to the microscope, and measured via a video microscaler (model VPA 550, For-A-Corp., Los Angeles, CA). The ports attached to the cranial window ring fit 17-gauge hypodermic needles for CSF sampling, washout, and drug administration. Cortical periarachnoid CSF was collected at baseline and 60 min after HI for ERK activity analysis.

### HI Induction

Hypoxia was induced for 10 minutes by switching room air to N_2_ for ventilation, followed by restoring ventilation to room air. Global cerebral ischemia was then induced by infusing saline through a hollow bolt in the cranium to maintain intracranial pressure higher than the mean blood pressure for 20 min. Global cerebral ischemia is confirmed when the blood flow in pial arteries were stopped visible on the monitor connected to the microscope over the cranial window. In order to avoid Cushing response (arterial pressure rising dramatically because of high intracranial pressure), blood was withdrawn when necessary to maintain the mean arterial blood pressure below 100 mmHg. The blood was returned via femoral vein at the end of ischemia.

### Drug Treatments

Four groups of i.v. drug administration were performed after HI (n = 5 in each group): (1) DMSO group: with DMSO (vehicle of salvinorin A) 1 µl/kg administrated immediately after HI; (2) SA 0 min group: with salvinorin A (1 µg/µl in DMSO) 10 µg/kg immediately after HI; (3) SA 30 min group: with salvinorin A (1 µg/µl in DMSO) 10 µg/kg 30 min after HI; (4) SA+Norbin group: with salvinorin A (10 µg/kg) and nor-BIN (1 µM, topically injected through one port of cranial windows) immediately after HI.

### Pial Artery Responses

Pial artery responses to hypercapnia, hypotension and isoproterenol (10 nM, 1 µM) were obtained before HI and 60 minutes after HI as previously described [16]. Isoproterenol was used as a positive control since it is a short acting agent and its vascular dilatation effect in such model is well established in our lab. Two levels of hypercapnia (PaCO_2_ of 50 to 60 mmHg for low level, 70 to 80 mmHg for high level) were produced by inhalation of high concentration CO_2_ mixture gas (10% CO_2_; 21% O_2_; 69% N_2_). Two levels of hypotension were produced by the rapid withdrawal of either 5–8 or 10–15 ml/Kg blood from the femoral artery to induce moderate and severe hypotension (25% decrease in mean blood pressure as moderate and 45% decrease as severe). Such decreases in blood pressure were maintained constantly for 10 min by withdrawal or reinfusion of additional blood.

### ERK Activity Measurement

ERK activity were then determined from frozen CSF samples described above. The levels of pERK and ERK were measured by ELISA kits (Enzo Life Sciences International, Inc., Plymouth Meeting, PA).

### Statistical Analysis

The data of pial artery diameter were analyzed by repeated-measures ANOVA followed by Bonferroni method as post hoc tests. One way ANOVA was used to compare the ERK activity changes (quantified as the ratio of pERK over ERK) in each group before and 60 min after HI and in the groups with or without salvinorin A administration. The baseline data were excluded in the repeated-measures analysis (Graph Pad Prism version 5.02). An alpha level of P<0.05 was considered significant in all statistical tests. Values are represented as means ± standard error.
